# Molecularly Imprinted Biodegradable Nanoparticles

**DOI:** 10.1038/srep40046

**Published:** 2017-01-10

**Authors:** Mariacristina Gagliardi, Alice Bertero, Angelo Bifone

**Affiliations:** 1 Istituto Italiano di Tecnologia, Center for Micro Bio-Robotics @SSSA, viale Rinaldo Piaggio,34, 56025, Pontedera, Italy; 2 Istituto Italiano di Tecnologia, Center for Neuroscience and Cognitive Systems @UNITN, Corso Bettini 31, 38068 Rovereto, Italy; 3 University of Pisa, Department of Biology, Unit of Cellular and Developmental Biology, S.S.12 Abetone e Brennero 4, 56127, Pisa, Italy

## Abstract

Biodegradable polymer nanoparticles are promising carriers for targeted drug delivery in nanomedicine applications. Molecu- lar imprinting is a potential strategy to target polymer nanoparticles through binding of endogenous ligands that may promote recognition and active transport into specific cells and tissues. However, the *lock-and-key* mechanism of molecular imprinting requires relatively rigid cross-linked structures, unlike those of many biodegradable polymers. To date, no fully biodegradable molecularly imprinted particles have been reported in the literature. This paper reports the synthesis of a novel molecularly- imprinted nanocarrier, based on poly(lactide-*co*-glycolide) (PLGA) and acrylic acid, that combines biodegradability and molec- ular recognition properties. A novel three-arm biodegradable cross-linker was synthesized by ring-opening polymerization of glycolide and lactide initiated by glycerol. The resulting macromer was functionalized by introduction of end-functions through reaction with acryloyl chloride. Macromer and acrylic acid were used for the synthesis of narrowly-dispersed nanoparticles by radical polymerization in diluted conditions in the presence of biotin as template molecule. The binding capacity of the imprinted nanoparticles towards biotin and biotinylated bovine serum albumin was twentyfold that of non-imprinted nanoparti- cles. Degradation rates and functional performances were assessed in *in vitro* tests and cell cultures, demonstrating effective biotin-mediated cell internalization.

Molecular Imprinting (MI) is a technique that endows a polymeric material with “molecular memory”^1^ by cross-linking functional monomers around a *template* molecule. Upon polymerization and removal of the template, binding sites complementary to the ligand^2^ in size, shape and chemical properties remain imprinted in the rigid polymer, thus enabling recognition and selective binding. While the use of MI in separation processes is widespread, applications in other fields, such as in targeted drug delivery, are still limited. Typically, target-recognition properties are built into nanoparticles by conjugation with targeting units, e.g. peptides decorating the surface of the nanoparticles. However, rapid enzymatic degradation of the targeting moieties in the biological milieu can strongly reduce the efficacy of this approach. Thus, completely synthetic Molecularly Imprinted Particles (MIPs), able to recognize and bind endogenous ligands, would be very attractive for applications in targeted drug delivery. This method does not require functionalization of the nanoparticles with a targeting unit, as molecularly imprinted sites enable the selective binding of the endogenous ligand, thus generating the desired functionality. Moreover, MIPs may promote active transport into specific tissues and cell types exploiting natural mechanism of cellular uptake and providing highly specific targeted drug delivery systems^3^,^4^,^5^,^6^,^7^,^8^,^9^. Despite their potential, the development of MIPs as carriers for drug delivery is still in its infancy^10^ and the simultaneous optimization of biocompatibility, delivery kinetics and molecular recognition properties remains a challenge^11^,^12^,^13^,^14^.

In the last few years, biodegradable polymers have attracted considerable interest for applications to drug delivery, owing to their biocompatibility and control of drug release. Indeed, biodegradation of polymeric components may prevent accumulation of the nanoparticles in organs and tissues, thus improving the safety profile of the material, while enabling fine control of release kinetics. To the best of our knowledge, to date, there is no proven synthetic strategy to obtain biodegradable MIPs with controlled size and shape. The literature reports very few studies on the preparation of biodegradable MI membranes^15^,^16^,^17^, or core/shell particles with biodegradable core and a stable imprinted shell^18^. However, practical demonstrations of targeted, fully biodegradable MIPs are still lacking. This reflects the difficulty to develop biodegradable materials with a sufficiently rigid cross-linked structure like that needed for the “lock and key” mechanism.

Here, we report the synthesis and characterisation of a novel PLGA-based biodegradable MIP system. Through the polymerisation of a three-arm acryloyl-terminated PLGA-based crosslinker, nanoparticles were imprinted toward biotin, an endogenous vitamin that induces receptor-mediated endocytosis. Three reactive sites per cross-linker molecule conferred sufficient stiffness to the nanoparticle structure for the imprinting, while ester bonds of PLGA segments ensured biodegradability. This study shows the first example of the application of the MI technology to full biodegradable nanoparticles.

## Results and Discussion

### Biodegradable cross-linker, synthesis and characterization

Starting from lactide (LA) and glycolide (GA) as monomers, and glycerol as initiator of the Ring-Opening Polymerization (ROP), the three-arm backbone of the biodegradable cross-linker was prepared. End-chain functionalization with acryloyl chloride introduced functional groups that, after radical reaction, gave cross-linked MIPs. Detailed synthesis procedures are reported in the Methods section. GPC analysis confirmed the desired molecular weight (8 kDa, Supplementary Fig. S1a), and provided a non-linear Mark-Houwink plot (Supplementary Fig. S1b), typical for nonlinear macromolecules, confirming that ROP is effective also in branched polyesters. The molar composition of PLGA chains was evaluated from 1H-NMR spectra (Supplementary Fig. S2a), and the resulting LA/GA molar ratio was 49/51, close to the fixed theoretical value (50/50). 13C-NMR (Supplementary Fig. S2b) and FTIR (Supplementary Fig. S1c) confirmed the molecular structure and the end-chain functionalization with acryloyl chloride.

### MIPs, synthesis and morphological and chemicophysical characterization

**Figure 1:**
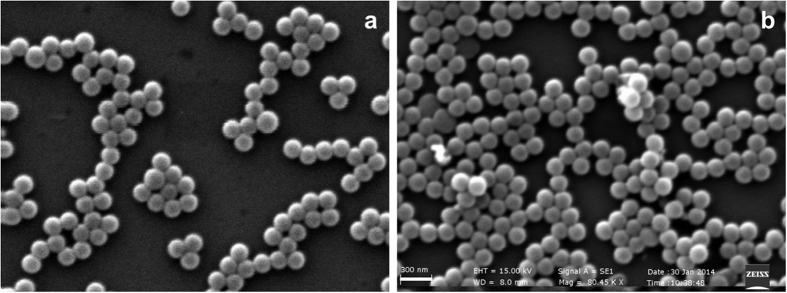
SEM analysis: (**a**) MIPs (after template extraction) (**b**) CPs; scalebar: 300 nm.

MIPs and Control Nanoparticles (CPs) were prepared following the procedure described in Methods section. In the radical reaction, we added a small amount of acrylic acid that ensured the presence of additional functional groups in the final product, enabling conjugation with a fluorescent dye. Synthesis was performed in diluted conditions and without additional surfactants, obtaining monodispersed and spherical nanoparticles (Fig. 1 and Table 1).

As template, we selected biotin, a small molecule that can induce efficient receptor-mediated endocytosis^19^; moreover, growing evidence suggests a role for this compound in cell signalling, gene expression, and chromatin remodeling, together with its potential involvement in cancer cell proliferation^20^. Molecular imprinting with biotin did not affect the process of nanoparticle nucleation and growth in solution, a we found no evidence of change in shape, size or aggregation compared to control particles (CPs).

**Figure 2:**
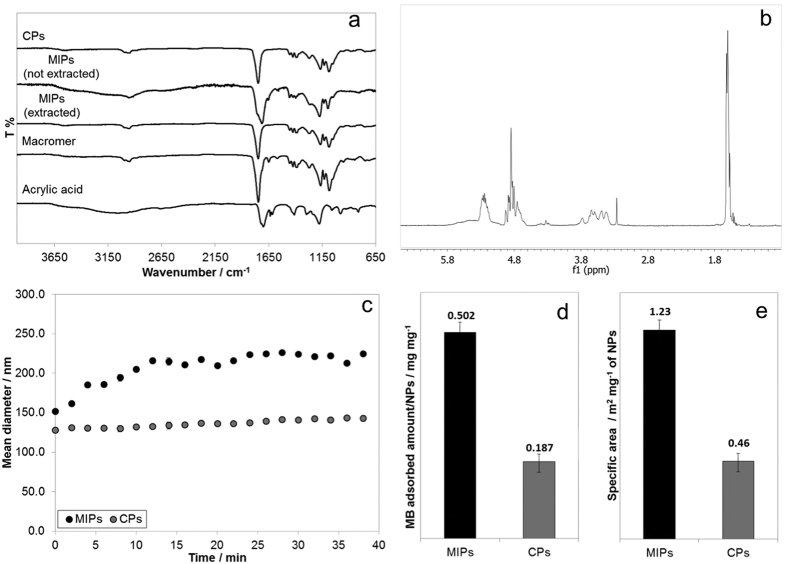
FTIR analysis (**a**) comparison between starting reactants and nanoparticles, and spectra of MIPs before and after template extraction; (**b**) 1H-NMR of extracted MIPs; (**c)** swelling tests by PCS; MB absorption tests: (**d**) amount of absorbed MB; (**e**) specific surface of nanoparticles.

The comparison of FTIR spectra of MIPs nanoparticles, before and after template extraction (Fig. 2a), demonstrated the efficacy of the template-removal procedure. In particular, the characteristic signals of the carboxylic and amine groups of biotin, detected between 3100 cm^−1^ and 3600 cm^−1^ (O-H and N-H stretching, broad signal) and at 1700 cm^−1^ (C = O stretching, overlapped to the signal due to carbonyl of polyester bonds) did not appear in the spectra from extracted nanoparticles. Moreover, the 1H-NMR spectrum (Fig. 2b) of MIPs confirmed the presence of both PLGA and acrylic acid in the imprinted nanoparticles and the complete extraction of the template molecule.

Swelling tests (Fig. 2c) showed that particles dispersed in water increased their diameter in the first 40 min, as a consequence of water uptake. The swelling degree (see Eq. (1) in Supplementary Information) was around 0.5 for MIPs and 0.1 for CPs at the end of the test. In particular, MIPs were more hydrophilic than CPs, due to the higher level of nanoporosity of MIPs resulting from the imprinting process. This hypothesis was confirmed by methylene blue (MB) absorption tests: results reported in Fig. 2d and e show that the overall MIP surface area was 2.7 times larger than that of CPs.

### Recognition properties

**Figure 3:**
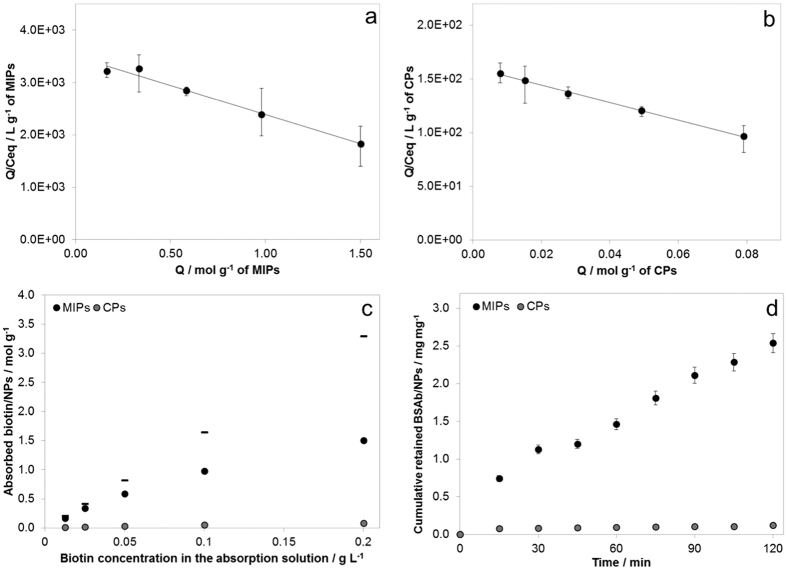
(**a**) Scatchard analysis on a MIPs and (**b**) CPs nanoparticles; (**c**) absorbed amount of biotin at different starting concentrations *vs* maximum amount (indicated by -); (**d**) rebinding tests on BSAb.

The dissociation constant (kd) and the maximum amount of bound ligand ([Qmax]) were estimated from the Scatchard analysis (Fig. 3a,b and Table 2). The linear trend of the Scatchard plot denotes the presence of one kind of binding sites, while nonlinear profiles are generally observed in MI systems, indicating the presence of binding sites with various affinities to the ligand^21^. Notably, MIPs showed 20-fold higher maximum biotin sorption capacity than CPs. Furthermore, the lower value of kd for MIPs compared to CPs indicates a higher stability of the biotin/nanoparticle complex formed after rebinding, thus confirming the effect of molecular imprinting on recognition and retention properties.

Rebinding tests with biotin-conjugated bovine serum albumin (BSAb) were also performed to assess the ability of MIPs to recognize and bind larger molecules functionalized with the template^22^,^23^. The rebinding kinetic profiles (Fig. 3d) demonstrated significantly higher recognition of the biotinylated albumin by MIPs than CPs (Table 3). The imprinting factor, defined as the ratio between the rebinding capacity of MIPs and the nonspecific sorption by CPs, was around 21, in line with results obtained with biotin alone.

While binding sites are also present inside the structure of MIPs, we can hypothesize that the protein was mainly bound by recognition sites exposed on the nanoparticle surface. Indeed, due to its size (Mw = 66 kDa^24^) and steric hindrance, albumin cannot diffuse into the polymer to reach the binding sites inside the nanoparticles.

We have evaluate the affinity distribution of binding sites at different biotin concentration, following the method reported by Tse Sum Bui and Haupt^25^. Trends of bound (*B*) and free (*F*) biotin concentrations and the number of binding sites *N* for MIPs and CPs are summarized in 4a-b. Data confirmed that binding sites populating MIPs are dependent on the association constant *K*, with *N* that ranges from 220 to 290 mol g^−1^ of MIPs, while for non-imprinted particles *N* is not dependent on the same parameter, with an almost constant value around 55.

**Figure 4:**
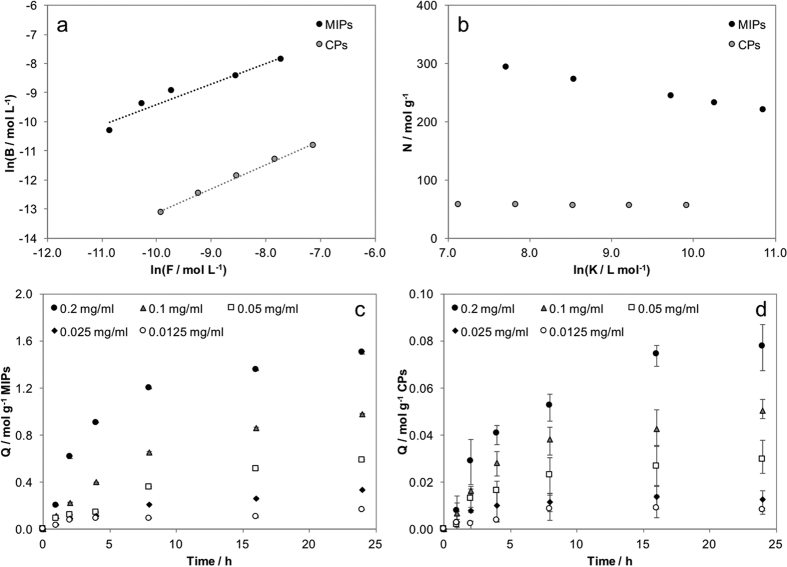
Evaluation of affinity distribution (**a**) bound (B) *vs* free (F) concentration in isothermal sorption tests at different starting biotin concentration (**b**) distribution of binding sites as a function of the association constant K; Sorption kinetics of biotin at 37 °C for (**c**) MIPs and (**d**) CPs nanoparticles.

The biotin sorption kinetics for MIPs and CPs, at different starting concentration of biotin and at fixed temperature (37 °C) was finally evaluated. Results, summarized in Fig. 4c,d, underline the higher sorption capability of MIPs in comparison with CPs.

### Degradation tests

Nanoparticle degradation tests were performed by PCS^26^,^27^ and GPC^28^. The results of PCS, however, were affected by the presence of nanoparticle fragments in the degradation medium and we could not accurately determine degradation rates. A more reliable analysis, taking into account the fractioning of nanoparticles, was performed by GPC.

Commonly, in the GPC analysis of nanoparticles, the apparent molecular weight is derived from a calibration curve obtained from standards with known molecular weights; this procedure is, in some cases, unreliable for the different molecular arrangement of the linear standards with respect to solid nanoparticles. Here, we have used of a calibration curve based on solid polystyrene nanoparticles with known diameters to overcome this limitation. This method allowed estimating nanoparticle diameters as a function of degradation time.

**Figure 5:**
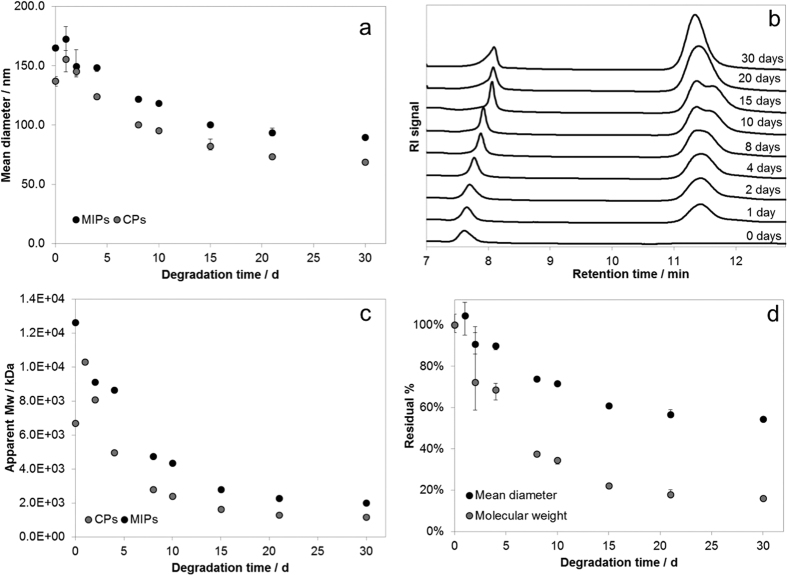
Degradation tests by GPC data: (**a**) mean diameter decrease *vs* time; (**b**) chromatograms for MIPs; (**c**) apparent molecular weights of degraded nanoparticles; (**d**) residual % of diameter and molecular weight of MIPs.

Results of degradation tests on MIPs and CPs (Fig. 5a) showed a decrease in mean diameter over time. In particular, the residual diameters of nanoparticles after 30 days were 54.4% and 50.3% for MIPs and CPs, respectively. GPC traces (Fig. 5b) showed the peak of nanoparticles at 7.5–8.0 min and an additional peak at late retention times, between 11 min and 12 min, indicating the presence of very small solid fragments and oligomers. An estimate of apparent molecular weights of cross-linked nanoparticles was obtained by fitting the Mark-Houwink-Sakurada (MHS) equation to the data (Fig. 5c). Residual diameter and apparent molecular weight percent for MIPs are compared in Fig. 5d.

Literature reports show slower degradation kinetics for PEG-PLA nanoparticles with smaller diameter (residual weight 63% after one month)^29^ but also for nanoparticles composed of PLGA (residual weight 40%) or mPEG-PLGA (residual weight 60% after one week)^30^ of comparable diameters and under similar conditions. The faster degradation kinetics of our nanoparticles can be attributed to the high porosity of their structure, the enhanced degree of hydrophilicity, and the low molecular weight of PLGA segments.

### Enhanced internalization capability

**Figure 6:**
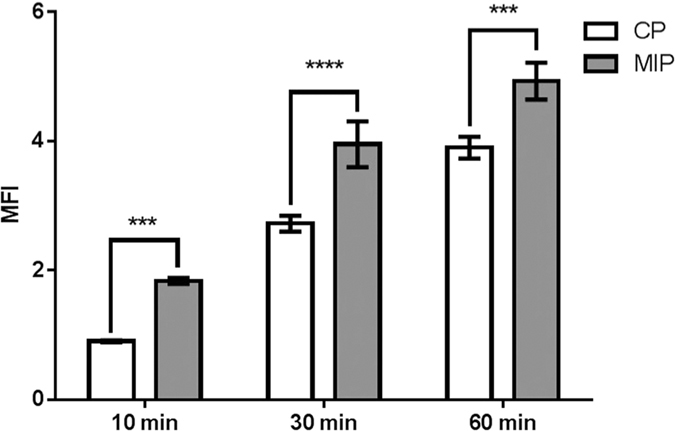
Nanoparticle internalisation into Hela cells. Bar graphs represent the mean fluorescence intensity (MFI) of HeLa cells treated with control nanoparticles and MIP-nanoparticles; data are expressed as mean ± SE of at least three independent experiments; statistical significance versus controls was calculated using one-way ANOVA with Dunnett’s post hoc test (***p < 0.001, (****p < 0.0001).

The biotin recognition capabilities of our MI system make it a potential candidate as carrier for intracellular delivery with improved internalization properties. Since an over-expression of biotin receptors on the membrane of various cancer cells has been observed, MIPs against biotin may be exploited for selective targeting^31^,^32^. As preliminary proof-of-concept of the functional properties of biotin-imprinted NPs we quantified the difference in biotin-receptor binding on fluorescently labeled MIPs and CPs by Flow Cytometry at different time points (Fig. 6). Biotin was present in the foetal bovine serum supplemented in HeLa cell tissue culture. Relative fluorescence intensity of HeLa cells almost doubled after 10 minutes from MIPs administration compared to CPs. The difference between the two nanoparticles internalization rate reached a maximum in 30 minutes and was still statistically significant after 60 minutes. During the first 30 minutes, receptor-mediated internalization of MIPs caused a rapid and specific uptake of imprinted nanoparticles, while CPs were internalized by passive endocytosis. These data suggest that bound biotin was still available for receptor mediated cellular uptake and the complex MIPs/biotin was able to efficiently penetrate cells that overexpress biotin receptor.

## Conclusions

Molecularly imprinted polymer nanoparticles have potential for the preparation of carriers for targeted drug delivery. Here, for the first time, the MI technology was applied to the preparation of fully biodegradable nanoparticles. The combination of biodegradation, molecular recognition and binding properties paves the way to the development of completely synthetic MIPs, with potentially important advantages in terms of stability, biocompatibility and cost of the formulation. Our results demonstrate the synthesis of imprinted nanoparticles with good recognition and binding properties towards biotin, alone or conjugated to moieties with larger molecular weights.

Active molecules can be loaded into our nanoparticles by physical absorption or by chemical conjugation. It can be foreseen that swollen structure of the nanoparticle provides a diffusive control of drug loading and release kinetics; furthermore, the biodegradability at longer times ensures the complete release of the payload. Due to the relatively fast degradation of our MIPs, the drug could be also covalently bonded and released upon hydrolysis of the polymer chains. While these mechanisms will be explored in future work to further develop this material, the present results demonstrate the feasibility of biodegradable molecular imprinted particles with recognition and selective binding properties; this is the first step in the exploitation of MI technology for the preparation of biodegradable nanoparticles for targeted drug delivery systems based on completely synthetic and biodegradable carriers.

## Methods

### Synthesis of the biodegradable crosslinker

Crude monomers lactide and glycolide monomers (LA and GA, Sigma Aldrich) were precipitated in ethyl acetate (Sigma Aldrich, Chromasolv^®^) and exsiccated at room temperature under vacuum; glycerol (Gly, Sigma Aldrich) was used as as initiator and Tin(II) 2-ethylexanoate (Oct, Aldrich) as catalyst. Acryloyl chloride (AC, Aldrich), 4-(Dimethylamino)pyridine (DMAP, Aldrich), triethylamine (TEA, Sigma Aldrich) and dichloromethane anhydrous (DCM, Sigma Aldrich) were used for end-chain functionalization. Acetone, methanol and diethyl ether (Sigma Aldrich, ACS purity degree) were used as solvents.

Monomers (60 mmol of LA, 60 mmol of GA) and initiator (2 mmol of Gly, corresponding to 6 mmol of -OH groups) were inserted in a round-bottom single-neck glass reactor and heated at 180 °C; when temperature was reached, the catalyst (1.2 mmol) was added; the reactive mass was dried under N2 using a CaCl2 column. With this recipe, the theoretical molar ratio LA/GA of copolymer chains was 50/50 and the theoretical molecular weight of the final product 8 kDa. The reactive mass was maintained at 180 °C for 3 h under mild stirring (100 rpm), then the reactor was quenched in a water bath, the solid product was dissolved in acetone (20 ml) and precipitated in methanol (50 ml) three times and finally dissolved in acetone (20 ml) and precipitated in diethyl ether (50 ml).

The end-chain functionalization of -OH terminal groups was performed dissolving the prepared product (12.5 of macromer, corresponding to 37.5 of -OH reacting groups) in DCM (20 ml) in a round-bottom two-necks glass reactor together with TEA (37.5); when the solution was homogenous, AC (45.0, with a slight excess with respect to -OH molar concentration) was added under stirring in an ice bath for 10 min; finally, DMAP (38.0) was added. Reaction was maintained under stirring, at room temperature, for 8 h. At the end, the product was concentrated with a rotary evaporator, precipitated three times in methanol and one time in diethyl ether, and then exsiccated for 24 h under vacuum. Product was stored at −20 °C in a glass vial.

### Synthesis of imprinted (MIPs) and control (CPs) nanoparticles

Nanoparticle synthesis involved the novel-synthesized biodegradable cross-linker and acrylic acid anhydrous (AA, Aldrich, 99%). AA was purified using a glass column packed with an inhibitor remover, then stored at −20 °C and used within one week. Acetonitrile (ACN, Sigma Aldrich, HPLC purity degree) and bidistilled water were used as porogen; potassium persulfate (KPS, Aldrich, ACS degree) was used as radical initiator, and biotin (Sigma Aldrich, >99%) as template molecule. The cross-linker (3.1) was dissolved in ACN (3 ml) in a glass reactor under mild stirring; AA (1.6) and biotin (40.9) were diluted in 0.5 ml of ACN separately and added to the reactor, then the temperature was increased to 70 °C; reactants and template molecule were maintained under stirring for 30 min before the introduction of the radical initiator, previously dissolved in 1 ml of distilled water at room temperature. An Allhin reflux condenser was used for the control of the reaction temperature; the reactor was flushed with N2 during the reaction. Control nanoparticles (CPs) were prepared with the same procedure, without the template molecule. Nanoparticles were purified by dialysis over water (5 h) and then over ethanol (2 h), and finally lyophilized and stored at −20 °C before the use. This procedure allowed elimination of unreacted monomers and initiator, and also extraction of the template molecule from MIPs.

### Recognition properties

Scatchard analysis was performed to evaluate equilibrium dissociation constant kd and maximum amount of retained biotin [Qmax]. The Scatchard analysis was described by the following Equation (1)^33^:


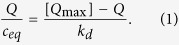


where *Q* is the amount of biotin retained by nanoparticles in mol g^−1^ and *c*_*eq*_ is the biotin concentration in the test solution in mol L^−1^. Analysis was performed on polymer nanoparticle samples (0.25 ± 0.05 mg) immersed in phosphate buffered saline (PBS) solution with pH 7.4 containing biotin at different concentrations (from 12.5 ml^−1^ to 200 ml^−1^) and maintained in a thermostatic bath at 37 °C throughout the duration of test period; after 24 h, samples were centrifuged at 14 krpm for 1 min, and the supernatant solution was collected and analysed by High-Performance Liquid Chromatography (HPLC) to evaluate the residual biotin not retained by nanoparticles. The same test was performed on CPs as reference to evaluate the nonspecific contribution to the sorption process. The chromatographic method used a Water chromatograph, a Luna C8 (Phenomenex) column maintained at 30 °C; ACN/water (50/50 v/v, isocratic, 1 ml min^−1^) was used as internal phase; data were collected from UV detector at 220 nm wavelength.

The affinity distribution was calculated with a graphical method, based on Freundlich isotherm, as reported in the literature^25^. Briefly, the trend of the concentration of bound (*B*) biotin as a function of free biotin (*F*) can be described with a power law:


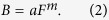


Experimental data were reported in a logarithmic plot (Fig. 4a) and fitted, obtaining *a* and *m*. From *a* and *m*, values of *N* were evaluated as:


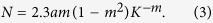


while association constants *K* for each free biotin concentration were evaluated as:


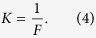


Affinity distribution, given by the trend of *N* with *ln(K*), for MIPs and CPs are summarized in Fig. 4b.

Sorption kinetics onto MIPs and CPs was evaluated with the same procedure but samples were collected and analyzed at 1, 2, 3, 4, 8, 16 and 24 h after the test was started. Rebinding tests were performed on both MIPs and CPs nanoparticles to evaluate the capability to recognize the biotin molecule after extraction. Tests were carried out by immersing extracted MIPs nanoparticles and CPs (1.64 ± 0.28 mg) in 0.5 ml of biotinylated bovine serum albumin (BSAb, Sigma) solutions (150 ml^−1^); samples were withdrawn at different times (from 0 to 120 min) and centrifuged, supernatant was collected and analysed by UV-vis spectrophotometer (V550 spectrophotometer, JASCO Europe); values of absorbance at 280 nm were used for quantification of BSAb re-bound by nanoparticles.

### Degradation tests

Nanoparticle degradation tests were performed in triplicate maintaining samples (1 mg) in PBS at 37 °C for the test period. At different time points samples were collected and characterized by PCS and GPC. For PCS analysis, samples were inserted into plastic cuvettes and measured with a Zetasizer S90 Nano apparatus; for GPC analysis, samples were injected in an apparatus composed of a Shimadzu chromatograph and a multi-detector suite (Agilent, MDS 1260) equipped by UV detector, refractive index, viscometric detector and dual-angle light scattering (15° and 90°). Two columns, Aquagel-OH 40 and Aquagel-OH 30 (Agilent) were used for the analysis. The mobile phase was a 10% SDS solution in water; GPC measurements were performed at 40 °C.

### Enhanced internalization capability

Internalisation tests were performed with fluorescence-marked nanoparticles (see Supplementary Information).

Cell culture tests were performed to evaluate internalisation capabilities of MIPs compared to CPs. Cells were cultured in high glucose DMEM with 10% FBS, 2 mM L-glutamine, 1000 U ml^−1^ penicillin and 1 mg ml^−1^ streptomycin. Cells (passage 2–20) were maintained in a humidified cell culture incubator at 37 °C and 5% CO2. About 24 h after being plated at 60% confluence, HeLa cells were treated with fluorescent CPs or MIPs at a final concentration of 2 mg ml^−1^. Cells were harvested after trypsinisation at 10, 30 and 60 min of incubation with nanoparticles, and analysed by flow cytometry using a MACSQuant analyser (Miltenyi Biotec, Bergisch Gladbach, Germany) for nanoparticles internalisation.

## Additional Information

**How to cite this article**: Gagliardi, M. *et al*. Molecularly Imprinted Biodegradable Nanoparticles. *Sci. Rep.* **7**, 40046; doi: 10.1038/srep40046 (2017).

**Publisher's note:** Springer Nature remains neutral with regard to jurisdictional claims in published maps and institutional affiliations.

## Supplementary Material

Supplementary Information

## Figures and Tables

**Table 1:** Mean diameters (nm) evaluated by Scanning Electron Microscopy (SEM) and Photon Correlation Spectroscopy (PCS) (PDI in parentheses); SEM pictures were elaborated by using the ImageJ software.

	SEM	PCS
MIPs	147.4 ± 17.4	151.9 (0.020)
CPs	131.4 ± 32.6	127.9 (0.162)

**Table 2:** Dissociation constants kd and maximum amount of bound ligand per nanoparticle weight unit [Qmax] of MIPs and CPs systems; R2 is related to the goodness of fitting.

	MIPs	CPs
kd/mol L^−1^	8.97 · 10^−4^	1.23 · 10^−3^
[Qmax]/mol g^−1^	3.14	0.20
R2	0.94	0.99

**Table 3:** Characteristic parameters of MIPs compared to CPs evaluated at the end of rebinding tests with BSAb; rebinding capacity is expressed in µmol of BSAb over g of nanoparticles.

	MIPs	CPs
% BSAb retained	2.36 ± 0.80	1.01 ± 0.23
Rebinding capacity	38.06 ± 9.65	1.80 ± 0.02
