# Supplementary material for: Molecularly Imprinted Biodegradable Nanoparticles
Source: Sci Rep. 2017 Jan 10;7:40046. doi: 10.1038/srep40046 (PMC5223160; doi:10.1038/srep40046)
Supplement: Supplementary Information [file srep40046-s1.pdf]

Molecularly Imprinted Biodegradable Nanoparticles

Mariacristina Gagliardi[1,*], Alice Bertero[2,3], and Angelo Bifone[2]

[1]Istituto Italiano di Tecnologia, Center for Micro Bio-Robotics @SSSA, viale Rinaldo Piaggio,34, 56025, Pontedera, Italy
[2]Istituto Italiano di Tecnologia, Center for Neuroscience and Cognitive Systems @UNITN, Corso Bettini 31, 38068 Rovereto, Italy
[3]University of Pisa, Department of Biology, Unit of Cellular and Developmental Biology, S.S.12 Abetone e Brennero 4, 56127, Pisa, Italy
[*]mariacristina_gagliardi@hotmail.it

SUPPLEMENTARY INFORMATION

Chemicophysical characterisation of the PLGA-based cross-linker.

Molecular weight analysis was performed by Gel Permeation Chromatography with a GPC apparatus composed of a Shimadzu chromatograph, two ResiPore/PLGel GPC column (Agilent Technology, Italy), a tetra-detector composed of UV diode array, refractive index, light scattering and viscometer; THF (Sigma Aldrich, Chromasolv, 1 ml min^{-1}) was used as eluent; polymer samples were dissolved in THF (0.1 - 0.2 mg ml^{-1}) and injected (injection volume was 50 µL); a calibration curve obtained with narrow polystyrene standards was used for data analysis.
GPC results (Fig. S1a): M_n = 8.3 kDa, M_w = 10.8 kDa, M_p = 8.1 kDa. PDI = 1.30.
FTIR-ATR analysis (Fig. S1c) was conducted on thin film-shaped samples obtained by casting from acetone using a Cary 630 FTIR (Agilent Technology, Italy).
FTIR for the macromer backbone / cm^{-1}: 1383 and 1452 (bending of -CH_3 lactide units), 1747 (C=O stretching), 1271 (C-O ester stretching), 3520 (end-chain O-H stretching), 2864-3034 (C-H stretching of aliphatic chains). FTIR for the functionalized macromer / cm^{-1}: 1653 and 1575 (C=C stretching), the signal at 3520 cm^{-1} disappeared. NMR spectra were acquired on polymer solutions (2% w/v) in $CDCl_2$ (Aldrich) with a Bruker 400 MHz spectrometer (Bruker, Italy).
^{1}H-NMR (400 MHz, $CDCl_3$): δ / ppm 5.23 (-OC**H**(CH_3)COO-), 4.83 (-OC**H**$_2$COO-), 4.2-4.5 (-C**H**$_2$OC**H**O-), 1.59 (-OCH(C**H**$_3$)COO-) (Fig. S2a); for acryloyl end-functionalities, signals of geminal protons overlap with signals in the range 4.5-4.9 ppm, while the signal due to the COOC**H**=CH_2 proton appeared at 6.7 ppm. ^{13}C-NMR (400 MHz, $CDCl_3$): δ / ppm 16.67 (-O**C**H(CH_3)COO-), 60.61 (-O**C**H_2COO-), 69.11 (-OCH(**C**H_3)COO-), 66.55 (-**C**H_2OCHO-), 169.29 (-OCH(CH_3)**C**OO-), 166.46 (-OCH_2**C**OO-), 139.13 (-**C**H=CH_2), 106.43 (-CH=**C**H_2) (Fig. S2b).
^{13}C-NMR and FTIR confirmed the molecular structure and the end-chain functionalization with acryloyl chloride. LA/GA ratio was evaluated from values of the integrals of the peaks at 5.23 ppm and 4.83 ppm.
Thermal characterisation was performed by Differential Scanning Calorimetry (DSC) using DSC1 equipment (Mettler Toledo, Italy); samples (5-8 mg) were inserted in Al sealed capsules with punched lid, measures were conducted under N_2 atmosphere (80 ml min^{-1}), ramp temperature was 10°C min^{-1} in the range from -60°C to 180°C, data were collected from the second heat scansion. T_g were 37.8 ± 0.1°C and 30.5 ± 1.2°C, for the backbone and the end-functionalized macromer respectively (Fig. S1d). All tests were performed in triplicate.

Nanoparticle characterisation.

Morphological and dimensional analyses were performed by Scanning Electron Microscopy (SEM, Merlin UltraPluss, Zeiss) and Photon Correlation Spectroscopy (PCS, Zetasizer S90 Nano, Malvern).
Samples for SEM analysis were prepared dispersing nanoparticles in ACN and then shedding one drop on a glass support. The solvent was removed by low-pressure evaporation for 1 h, then sputtered with Au.
Samples for PCS analysis were prepared dispersing nanoparticles in methanol by ultrasonication for 3 min (1 mg ml^{-1}); subsequently different aliquots of dispersion were added to 1 ml of distilled water, equilibrated for 30 min and analysed (dual-angle analysis, 90° and 13°) to evaluate the aggregation index. Results were dependent on nanoparticle concentration, with more concentrated samples that showed aggregation. Data acquired from samples containing 0.2 mg ml^{-1} of nanoparticles showed an aggregation index close to zero.

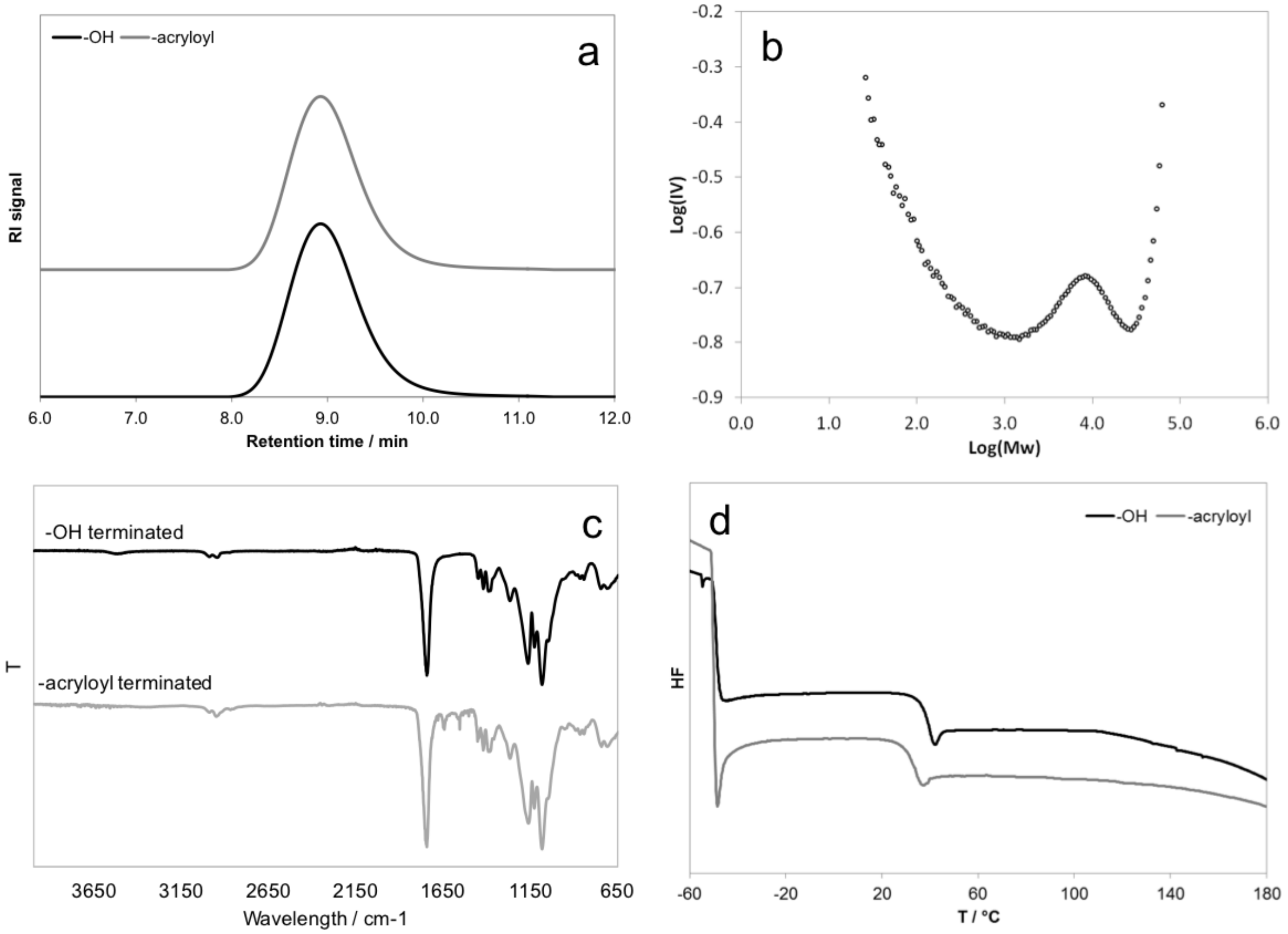

Figure S1. **a** GPC traces of PLGA-based backbone and end-functionalized crosslinker; reported data were acquired from RI detector; **b** Mark-Houwink plot obtained for the macromer backbone; **c** FTIR spectra of –OH and acryloyl-terminated macromer; **d** Thermograms of –OH and –acryloyl terminated macromer.

Swelling properties at early time-points (up to 40 min after the immersion of nanoparticles in aqueous medium) were evaluated on MIPs and CPs by PCS. Nanoparticles were dispersed in methanol (1 mg ml^{-1}) then diluted in water (10 µL in 1 ml) and inserted in a plastic cuvette suitable for the analysis. Samples were maintained at 25°C during the analysis and data were collected every 2 min for the entire test period. The swelling degree (SD), was evaluated as in Equation (1):

$$SD = \frac{d(t) - d(0)}{d(0)} \tag{1}$$

where $d(t)$ and $d(0)$ represented the mean diameters at time t and 0, respectively. All tests were performed in triplicate. The specific surface area of particles was evaluated through the methylene blue (MB) absorption test. Tests were performed on 1 mg of nanoparticles immersed in 1 ml of MB solution (2.5 µmol ml^{-1}) in methanol/water (10/90 v/v) for 24 h at room temperature. At the end of the test, samples were centrifuged and the supernatant was analysed by UV-vis (IRAffinity, Shimadzu, Italy) at 665 nm. Data were elaborated on the basis of a calibration curve obtained in the range of concentration of MB between 0.5 µmol ml^{-1} and 32.0 µmol ml^{-1}. The specific surface (A_s) of nanoparticles was evaluated through Equation (2)[1]:

$$A_s = \frac{q_{MB} \cdot N_A \cdot \phi_{MB}}{M_{MB} \cdot m} \tag{2}$$

where q_{MB} is the amount in g of absorbed MB, N_A is the Avogadro number ($6.022 \cdot 10^{23}$ mol^{-1}), ϕ_{MB} is the surface area

that a single MB molecule occupies on the adsorbent surface ($1.3 \cdot 10^{-18}$ m^2), M_{MB} is the molecular weight of MB (319.86 g mol^{-1}) and m is the mass of nanoparticles used for the test in mg. Five samples of MIPs and five of CPs were tested.

Synthesis of polystyrene nanoparticle standards.

Polystyrene nanoparticle standards (PNS) were synthesized by emulsion polymerisation in 50 ml of water, sodium dodecyl sulphate and ethanol (water+SDS/EtOH) (4/1 v/v, 10% w/v SDS in the water phase) by varying the monomer concentration to tune nanoparticle diameters. Monomer concentration was varied between 1% and 12% v/v; potassium persulfate (1% mol/mol in respect to the monomer concentration) was used as initiator, and the reaction was performed at 70°C for 4 h. At the end of the reaction PNS were filtered over a paper filter and washed several times with hot water (50°C) to eliminate residual monomer, SDS and the initiator. Subsequently NPS were washed with EtOH and exsiccated under vented hood overnight. Morphological characterisation of PNS was performed by PCS and SEM following the same procedure described for MIPs and CPs. After the evaluation of diameters and polydispersity by PCS, PNS were injected into the GPC apparatus and a correlation of diameters vs retention time was measured.

Calibration curve for degradation tests.

The calibration curve was obtained analysing PNS. Mean retention times were plotted as a function of the mean radius, evaluated by PCS (Fig. S3).

The multi-detection analysis allowed evaluating retention times and apparent Mark-Houwink-Sakurada plots. The resulting MHS constants α and k were $\alpha_{\mathrm{PS}} = 0.428$, $k_{\mathrm{PS}} = 7.67 \cdot 10^{-5}$ ml g^{-1}, $\alpha_{\mathrm{MIP}} = \alpha_{\mathrm{CP}} = 0.66$ and $k_{\mathrm{MIP}} = k_{\mathrm{CP}} = 9.96 \cdot 10^{-4}$. Molecular weights of PNS were evaluated by Equation (3) as described in Ref.[2].

$$ln(M_{MIP}) = \frac{1+\alpha_{PS}}{1+\alpha_{CP}} \cdot ln(M_{PS}) + \frac{1}{1+\alpha_{MIP}} \cdot ln\left(\frac{k_{PS}}{k_{MIP}}\right) \tag{3}$$

with

$$M_{PS} = \frac{\pi \cdot N_A \cdot \rho}{6} \cdot \left(\frac{d}{10^7}\right)^3 \tag{4}$$

where M_{PS} is the molecular weight of polystyrene nanoparticles, ρ the density (1.05 g cm^{-3}) and d the diameter evaluated by DLS.

Conjugation of fluorescent dye.

ATTO-633-NH2 (Atto-tec, Germany) was covalently bonded to carboxylic groups through acrylic acid moieties. Chemical conjugation was obtained on 10 mg of nanoparticles dispersed in CH_2Cl_2 anhydrous (5 ml) containing 0.42 µmol of EDCl; 100 µg of fluorescent dye (0.12 µmol) dissolved in 50 µL of DMSO were added to the reactor, together with 0.42 µmol of DMAP. Reaction was maintained at room temperature under stirring in the dark for 24 h. Nanoparticles were purified by osmosis over acetone (1 h), water (3 h) and finally ethanol (3 h). The volume of the dialysis medium was 50 ml, acetone was replaced every 15 min while water and ethanol every 30 min. The conjugation degree was evaluated through spectrofluorimetry on three samples. 25 µg of conjugated nanoparticles were dispersed in 1 ml of ethanol and inserted in a quartz cuvette. Results were elaborated on the basis of a calibration curve obtained by dissolving the dye in ethanol in the range of concentration 0.03-1.12 µg ml^{-1}. The degree of conjugation of MIPs resulted to be 0.23% ± 0.09%, expressed as percent µg of dye/µg of nanoparticles.

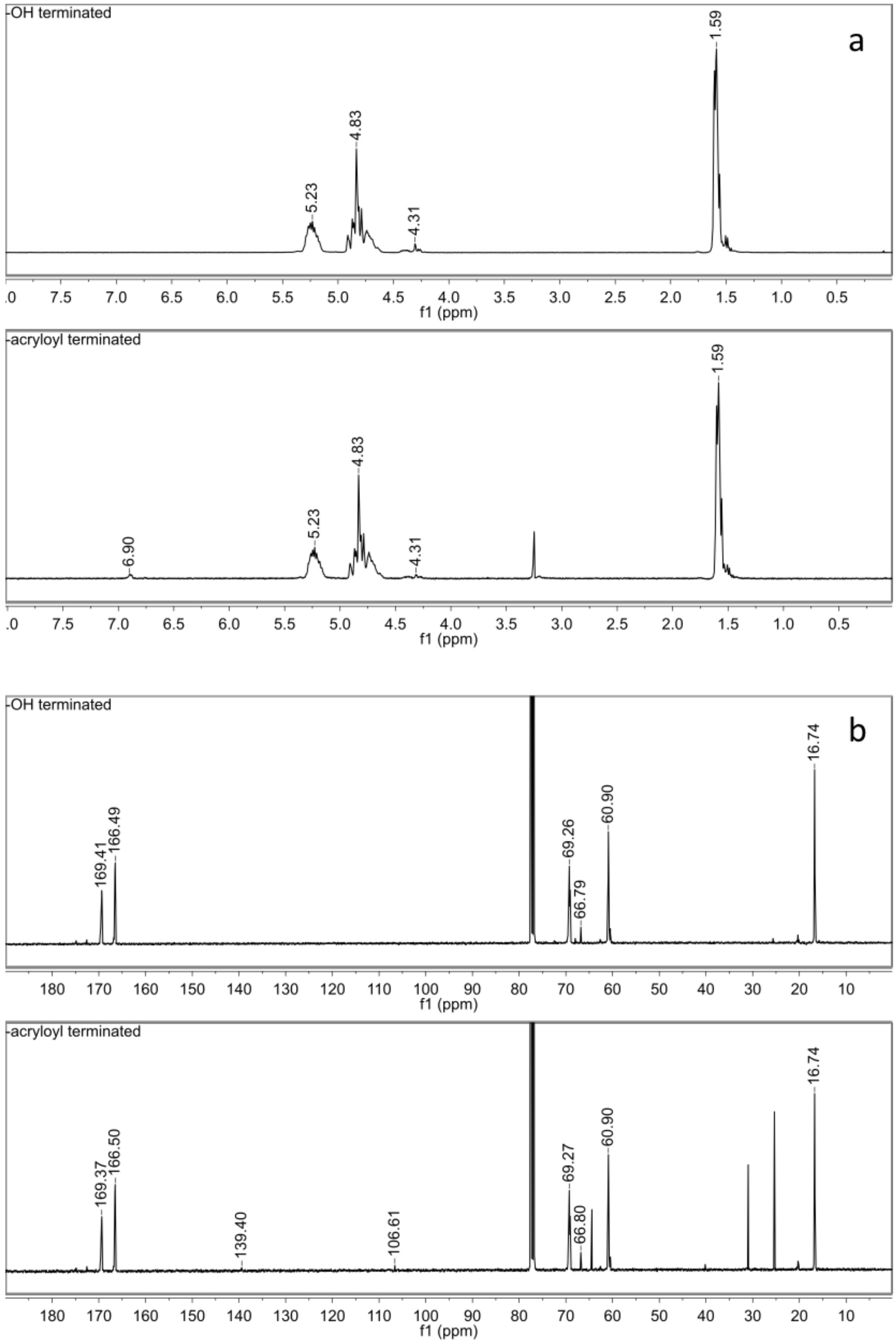

Figure S2. **a** ^{1}H-NMR spectra of –OH and acryloyl-terminated macromer; the signal due to $CDCl_3$ at 7.26 ppm was suppressed, the signal around 3.2 ppm in the spectrum of –acryloyl terminated product was due to residual TEA; **b** ^{13}C-NMR spectra of –OH and acryloyl-terminated macromer.

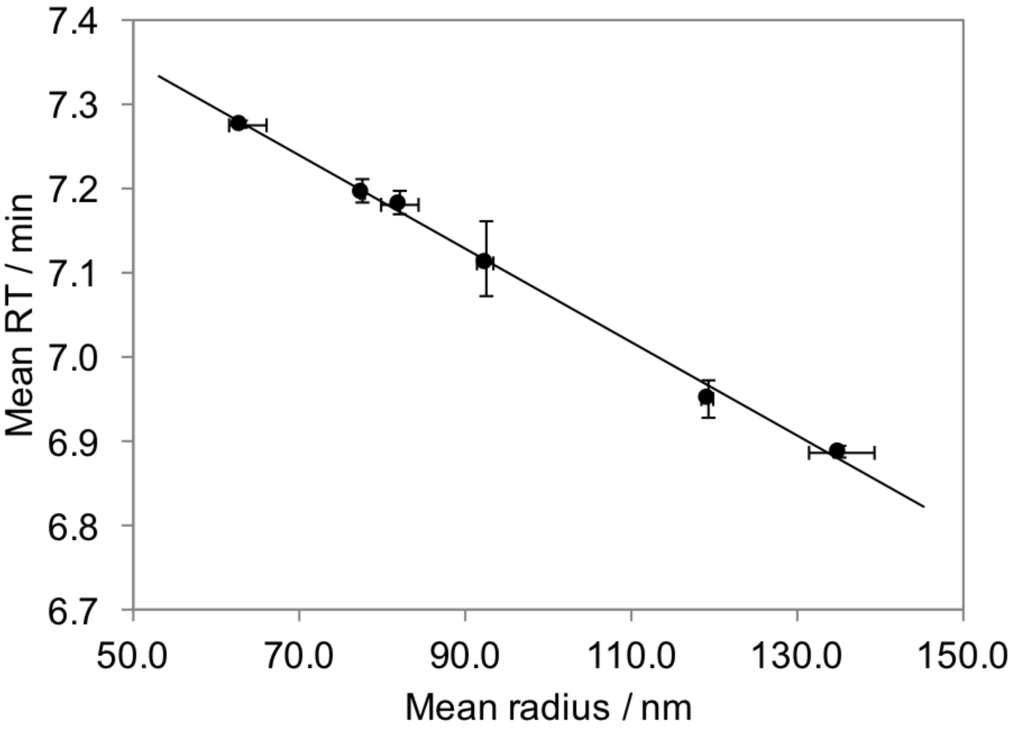

Figure S3. Calibration curve obtained on polystyrene nanoparticle standard.

References

1. Xu, D. *et al.* Rational design of molecularly imprinted photonic films assisted by chemometrics. *J. Mater. Chem.* **22**, 16572–16581 (2012).

2. Meunier, D. M. *et al.* Determination of particle size distributions, molecular weight distributions, swelling, conformation, and morphology of dilute suspensions of cross-linked polymeric nanoparticles via size-exclusion chromatography/differential viscometry. *Macromolecules* **47**, 6715–6729 (2014).
